# Organic and Polymeric Thin-Film Materials for Solar Cells: A New Open Special Issue in Materials

**DOI:** 10.3390/ma15196664

**Published:** 2022-09-26

**Authors:** Lung-Chien Chen

**Affiliations:** Department of Electro-Optical Engineering, National Taipei University of Technology, Taipei 10608, Taiwan; ocean@ntut.edu.tw

“Organic and Polymeric Thin-Film Materials for Solar Cells” is a new open Special Issue of *Materials*. Organic and polymeric semiconductors exhibit higher light absorption coefficients than their inorganic counterparts, to such an extent that they are suitable for photovoltaic applications to absorb light covering the entire solar spectrum, owing to the presence of π-bonded electrons [1].

The key success factors for organic and polymeric solar cells include improved photon capture through energy gap engineering—especially in the strongest solar radiation ranging between 400 and 800 nm—and improved charge generation and transport through polymer morphology engineering, as it is now very clear that the photo-induced charge separation is severely affected by the size of the donor/acceptor domain, and the charge mobility is severely affected by the morphology of the polymer. Solar energy stands out because it is environmentally friendly and not subject to geographical restrictions. Organic and polymeric solar cells have many competitive advantages, including convenient material chemical structure fine tuning, frontier orbitals, energy gap, material durability, as well as the low cost and versatility of solution-based, large-scale industrial processing and manufacturing, including sophisticated polymer solution printing technology or roll-to-roll processing protocols. Recently, a group of scientists from SCUT achieved a new record in a large-area organic solar module of 14.4% efficiency [2]. Therefore, to compare the inorganic semiconductor solar cells since the 1950s, the organic and polymeric semiconductor solar cells still have a great deal of room for development in the future. 

The areas of interest of the Special Issue “Organic and Polymeric Thin-Film Materials for Solar Cells” include, but are not limited to, the following: material, structure, process, modelling, characterization, simulation, and applications.
